# Supplementary material for: Mechanistic models of Rift Valley fever virus transmission: A systematic review
Source: PLoS Negl Trop Dis. 2022 Nov 18;16(11):e0010339. doi: 10.1371/journal.pntd.0010339 (PMC9718419; doi:10.1371/journal.pntd.0010339)
Supplement: S1 Text — Text A: Reading grid; Table A: Vaccination strategies implemented in models and main results; Table B: Characteristics of spatial models as well as non-spatial models with external renewal; Table C: Type of datasets and their use. (DOCX) Code used to produce figures and summary statistics is available in Github public repository at https://github.com/helenececilia/riftvalleyfever-model-review.git. [file pntd.0010339.s001.docx]

### **Text A: Reading grid**

#### *Research questions, context, main analyses and results*

- Main modeling objective (primary/secondary possible): understand; anticipate; control
- Model category: theoretical; applied; grey
- Geographical location: if applicable, precise if zone with history of RVF or RVF-free
- Context under study: hypothetical incursion or real world setting; endemic or epidemic scenario; other
- Geographical scale: international; national; sub-national; local
- Is a parent model clearly stated?
- What is common/different from the parent model?
- Indicators of disease spread: R (any type of reproductive ratio, precise if analytical, from data, by simulation); number of individuals in a given health state; summary statistic; other
- Model outputs: spatial patterns; temporal patterns; sensitivity of the model to parameters/hypothesis; parameter estimation; other
- Control measures tested?
- Model selection performed?
- Sensitivity analysis performed? If yes, which type: one-at-a-time; global
- Main results
- Limits of the model (as presented in the paper)

#### *Data*

If presence of data,

- Type of data: experimental; environmental; epidemiological; clinical; demographic; movement; other
- If the data spatialized, is it used as such in the model?
- If the data presented as time-series, is it used as such in the model?
- Use of data: calibration; model selection; input; inference/model assessment
- Origin of data: all published or open access; some are published for the first time in peer review

#### *Hosts*

- How many vertebrate taxa were considered?
- What taxa?
- What health states?
- Is there population renewal?
- Were age class considered? If yes, what type of age classes?
- Is the herd/human population detailed further (male/female; pregnant; occupation; other)?
- Important differences between host taxa or host types within a taxa : population density/dynamics; movement patterns; attractiveness to mosquitoes; infectiousness/susceptibility; clinical outcome; duration of latent/infectious period; development of immunity; some were dead-end hosts; other
- Clinical outcomes described : asymptomatic/symptomatic; mild/severe; disease-induced mortality; abortion; none/other

#### *Vectors*

- How was the vector component modeled: implicitly (no state variable); explicitly (at least one state variable)?
- How many taxa?
- What taxa?
- Main characteristics of the vector population and differences between taxa, precise when driven by abiotic factor: life cycle (emergence, feeding behavior); infection (probability, extrinsic incubation period); transmission (probability, vertical transmission)
- What health states?
- How was the aquatic population modeled: not at all; implicitly (emergence rate of adults described by a parameter or function); explicitly (at least one state variable); other (e.g. extracted from an external model)?
- If implicit aquatic population, type of parameter/function: constant; forced (e.g. sinusoidal, precise if informed by data); input-dependent (e.g. abiotic variable); other
- If explicit aquatic population, features (which state, influence of abiotic factors, possible dormancy etc.)
- Blood meal frequency: constant; variable (precise with what factor); not a parameter
- Blood meal distribution among hosts: homogeneous; heterogeneous; not a parameter
- Is there an explicit factor besides host taxa driving heterogeneous biting (host size, behavior, infection status etc.)?

#### *Spatiotemporal dynamics and modeling paradigm*

- How many spatial locations: one with no immigration or emigration/location was undefined or vaguely defined (spaceless model); more than one location or model included terms describing immigration or emigration?
- Type of spatial model: discrete number of locations/pixels; continuous space; connectivity network among individual hosts; other
- Resolution (surface covered by one pixel, if applicable)
- What moves: nothing; vertebrate hosts; vectors; both?
- If hosts move, what type of movement: commercial; nomadic; other?
- If vectors move, what type of movement: follow hosts; random; other (e.g. wind)?
- Duration of simulations, and if known, time period (month, year)
- Timestep
- Characteristics of the model: deterministic/stochastic; agent-based/compartmental
- If stochastic: what is stochastic, number of runs etc.

#### *Transmission and infection*

- Functional form of the force of infection (FOI, for vector-host transmission)
- Is the choice of functional form justified/discussed?
- Transmission parameter: aggregated, constant; aggregated with variation (function with no clear biological translation, e.g. proxy for seasonality); decomposed (different parameters for biological processes such as biting rate, probability of successful transmission, feeding preferences etc.)
- Other routes of transmission (precise FOI if applicable): mosquito-mosquito; livestock- livestock; mosquito-human (1 way or 2 ways); livestock-human (1 way)
- Hypothesis for time spent in E/I/R state, for hosts/vectors: exponential; other (precise if driven by abiotic factor)

**Table A: Vaccination strategies implemented in models and main results.**

| **Model** | **Category** | **Vaccine-related health state(s)** | **Time to build-up immunity** | **Efficacy** | **Coverage** | **Target specific hosts** | **Vaccination regimen** |
| --- | --- | --- | --- | --- | --- | --- | --- |
| Gaff et al. (2011) [1] | theoretical | explicit compartment : only from $S$ | Yes | Yes | Yes | No | periodic *vs* one-time *vs* constant |
| Main results: Most efficient strategy tested is preventive vaccination of 100% of livestock with 85% vaccine efficacy via a 28-day continuous campaign | | | | | | | |
| Adongo et al. (2013) [2] | theoretical | explicit compartment : only from $S$ | No | No | Yes (optimized in time through objective functional) | No | optimal (objective functional, no delay) |
| Main results: Optimal strategy varies with the weight put on cost-minimization (number of vaccinated) *vs* minimizing the infected class | | | | | | | |
| Chamchod et al. (2014) [3] | theoretical | $S\to R$ transition | No | No | Yes (optimized in time through objective functional) | No | optimal (objective functional, start with delay) |
| Main results: i) vaccinating at a strong rate before the outbreak is the most efficient strategy ii) if delay ≥ 2 months epidemic size is not decreased | | | | | | | |
| Chamchod et al. (2016) [4] | theoretical | explicit compartment ; Live vaccines : probability of reversion to virulence and possible abortions ; Killed vaccines : need of a booster to acquire lifelong immunity | Yes | Yes | Yes | No | periodic (start with delay) |
| Main results: i) introduction of ruminants vaccinated by live vaccines in RVFV-free areas may cause an outbreak and RVFV may become endemic if there is sustained use of live vaccines ii) if delay ≥ 3 months epidemic size is not decreased | | | | | | | |

**Table A - Continued**

| **Model** | **Category** | **Vaccine-related health state(s)** | **Time to build-up immunity** | **Efficacy** | **Coverage** | **Target specific hosts** | **Vaccination regimen** |
| --- | --- | --- | --- | --- | --- | --- | --- |
| Yang and Nie (2016) [5] | theoretical | explicit compartment : only from *S* | No | No | Yes | No | impulsive (length of cycle) |
| Main results: i) a short period of pulsing or a large pulse vaccination rate are sufficient conditions for the eradication of RVFV ii) RVFV is uniformly persistent if the vaccination rate is low and vaccination period is too long | | | | | | | |
| Gachohi et al. (2016) [6] | grey | unknown | Yes | Yes | Yes (reached progressively, not pulse event) | Yes (cattle *vs* small ruminants) | periodic *vs* reactive |
| Main results: i) similar coverage induce stronger reduction of incidence in small ruminants than in cattle ii) vaccinating cattle protects both cattle and small ruminants while vaccinating small ruminants only protects small ruminants iii) authors provide a relationship between level of reactive vaccination required to stop an outbreak, given a specific level of periodic vaccination implemented | | | | | | | |
| McMahon et al. (2014) [7] | grey | explicit compartment | Yes | Yes | No | No | reactive, with delay |
| Main results: For a 45-day rainy season, 3 week window of opportunity to vaccinate to impact ongoing epidemic. Otherwise, benefits of vaccination primarily occur in years subsequent to the vaccination effort | | | | | | | |
| Métras et al. (2020) [8] | applied | explicit compartment | Yes | Yes | Yes | Yes (livestock vs human) | reactive, mass vaccination, with  delay |
| Main results: i) vaccinating 20% of livestock immediately after first human case is reported reduces human cases by 30% ii) waiting one more month requires 50% more vaccine doses to achieve similar impact | | | | | | | |

**Table A - Continued**

| **Model** | **Category** | **Vaccine-related health state(s)** | **Time to build-up immunity** | **Efficacy** | **Coverage** | **Target specific hosts** | **Vaccination regimen** |
| --- | --- | --- | --- | --- | --- | --- | --- |
| EFSA AHAW Panel et al. (2020 - Model 1) [9] | applied | explicit compartment | No | Yes | No (number of animals vaccinated per day until 100% is reached | No | continuous, starting before or after RVFV incursion |
| Main results: i) the vaccination is more effective when applied early before the start of the epidemic and quickly implemented throughout the population  ii) vaccine effectiveness has a minor effect on the reduction of infections iii) Vaccinating 200 to 2000 animals a day guarantees the epidemic is halted within 1 year, regardless whether vaccination starts before or after RVFV incursion. With 20 animals vaccinated per day, the epidemic continues for more than 2 years, and the whole population eventually gets infected. | | | | | | | |
| EFSA AHAW Panel et al. (2020 - Model 2) [9] | applied | reduce probability of transmission (vector to host and host to vector) | Yes | Yes | No (start by infected premises until 100% vaccinated) | No | reactive, without delay |
| Main results: vaccination in a 50-km radius around detected farms is among the most effective strategies (other, not involving vaccination, are compared) | | | | | | | |

**Table B - Characteristics of spatial models as well as non-spatial models with external renewal.**

Without any geographical context, we chose not to assign a scale to theoretical models.

| **Reference** | **Category** | **Location** | **Scale** | **Connection** | **Movement data / use of proxy** |
| --- | --- | --- | --- | --- | --- |
| **Spatial models** |  |  |  |  |  |
| Niu et al. (2012) [10] | theoretical |  |  | vectors and hosts move | None |
| Xue and Scoglio (2013) [11] | theoretical |  |  | hosts move | None |
| Xue and Scoglio (2015) [12] | theoretical |  |  | indirect through FOI | None |
| Wen et al. (2019) [13] | theoretical |  |  | hosts move | None |
| Python Ndekou Tandong et al. (2020) [14] | theoretical |  |  | hosts move | None |
| Gao et al. (2013) [15] | grey | Egypt and Sudan | international | hosts move | None |
| McMahon et al. (2014) [7] | grey | East Africa | international | indirect through FOI | None |
| Mpeshe et al. 2014) [16] | grey | Tanzania | sub-national | disconnected entities | None |
| Xiao et al. (2015) [17] | grey | Egypt and Sudan | international | hosts move | None |
| Sumaye et al. (2019) [18] | grey | Tanzania | sub-national | hosts move | None |
| Xue et al. (2012) [19] | applied | South Africa | sub-national | vectors and hosts move | vectors : wind  livestock : animals sold, number in feedlots  humans : distance, population, commuting and return rates |
| Xue and Scoglio (2013) [11] | applied | US | sub-national | vectors and hosts move | maximum percentage of animals moving, extensive agricultural setting |
| Barker et al. (2013) [20] | applied | US | sub-national | disconnected entities | None |

**Table B - Continued**

| **Reference** | **Category** | **Location** | **Scale** | **Connection** | **Movement data / use of proxy** |
| --- | --- | --- | --- | --- | --- |
| Fischer et al. (2013) [21] | applied | Netherlands | national | disconnected entities | None |
| Nicolas et al. (2014) [22] | applied | Madagascar | sub-national | hosts move | renewal practices of cattle breeders : trade, barter, number of animals, origin |
| Leedale et al. (2016) [23] | applied | Kenya and Tanzania | international | disconnected entities | None |
| Taylor et al. (2016) [24] | applied | East African Community | international | disconnected entities | None |
| Scoglio et al. (2016) [25] | applied | US | sub-national | hosts move | None |
| Sekamatte et al. (2019) [26] | applied | Uganda | sub-national | indirect through FOI | None |
| Cecilia et al. (2020) [27] | applied | Senegal | sub-national | disconnected entities | None |
| EFSA AHAW Panel et al. (2020 - Model 2) [9] | applied | Netherlands | national | indirect through FOI | None |
| Tennant et al. (2021) [28] | applied | Comoros archipelago (including Mayotte) | international | hosts move | expert knowledge of local veterinary services to inform prior distribution of movement parameters (start date, duration, trade estimates, infectious imports) |
| **Non spatial, open models** |  |  |  |  |  |
| Gil et al. (2016) [29] | grey | Egypt | national | hosts entry (importations) | None |
| Métras et al. (2017) [30] | applied | Mayotte | sub-national | hosts entry | unofficial trade |
| Durand et al. (2020) [31] | applied | Senegal | local | hosts in and out | survey of nomadic herders : main stops, dates |

**Table C - Type of datasets and their use.**

A number is assigned to each dataset in column “Type of data” and used in subsequent columns for readability.

| **Study** | **Type of data used** (numbers defined in this column are used in the following columns) | **Spatialized data** | **Time series data** | **Use of data** | | |
| --- | --- | --- | --- | --- | --- | --- |
|  |  |  |  | **Calibration** | **Input** | **Inference / model assessment** |
| Métras et al. (2017) [30] | **Environmental**  - Vegetation (1)  **Epidemiological**  - Serological (livestock) (2)  **Demographic**  - Livestock + age structure (3)  **Movement**  - Importation (4)  - Starting date of import + duration (5) |  | 1, 2 | 3, 4 | 1 | 2, 5 |
| Métras et al. (2020) [8] | **Environmental**  - Rainfall (1)  **Epidemiological**  - Serological (livestock) (2)  - Serological (human) (3)  - Incidence (human) (4) |  | 1, 4 | 2,3 | 1 | 2, 4 |
| Cecilia et al. (2020) [27] | **Environmental**  - Temperature (indirect) (1)  **Demographic**  - Livestock (2)  - Vectors (3)  **Other**  - Vector trophic preferences (4) | 1, 2, 3 | 1, 3 | 4 | 1, 2, 3 |  |
| Durand et al. (2020) [31] | **Environmental**  - Temperature (indirect) (1)  **Epidemiological**  - Serological (livestock, age structured) (2)  **Demographic**  - Livestock (3)  - Vectors (4)  **Movement**  - Nomadic movements (5) |  | 1, 2, 4 | 3, 4, 5 | 1, 4 | 2 |

**Table C - Continued**

| **Study** | **Type of data used** (numbers defined in this column are used in the following columns) | **Spatialized data** | **Time series data** | **Use of data** | | |
| --- | --- | --- | --- | --- | --- | --- |
|  |  |  |  | **Calibration** | **Input** | **Inference / model assessment** |
| Leedale et al. (2016) [23] | **Environmental**  - Temperature (1)  - Rainfall (2)  **Epidemiological**  - Outbreaks dates (3)  - Mosquito surveillance (infection) (4)  **Demographic**  - Livestock (5)  - Vectors (6) | 1, 2, 3, 4, 6 | 1, 2, 3, 4, 6 |  | 1, 2, 5 | 3, 4, 6 |
| Taylor et al. (2016) [24] | **Environmental**  - Temperature (1)  - Rainfall (2)  **Other**  - Social vulnerability (3) | 1, 2, 3 | 1, 2 |  | 1, 2, 3 |  |
| McMahon et al. (2014) [7] | **Environmental**  - Land use (1)  - Rainfall (2)  **Demographic**  - Livestock (3)  - Humans (4) | 1, 2, 3, 4 | 2 | 1, 2 | 3, 4 |  |
| Mpeshe et al. (2014) [16] | **Experimental**  - Temperature dependent survival rates (1)  **Environmental**  - Temperature (2)  - Rainfall (3) | 2, 3 | 2, 3 | 1 | 2, 3 |  |
| Fischer et al. (2013) [21] | **Environmental**  - Temperature (1)  **Demographic**  - Livestock (2)  - Vectors (abundance) (3)  - Vectors (dynamics) (4) | 2, 3 | 1, 4 | 4 | 1, 2, 3 |  |

**Table C - Continued**

| **Study** | **Type of data used** (numbers defined in this column are used in the following columns) | **Spatialized data** | **Time series data** | **Use of data** | | |
| --- | --- | --- | --- | --- | --- | --- |
|  |  |  |  | **Calibration** | **Input** | **Inference / model assessment** |
| Nicolas et al. (2014) [22] | **Environmental**  - Land use (1)  **Epidemiological**  - Serological (livestock) (2)  **Demographic**  - Livestock (density) (3)  - Livestock (calving and mortality) (4)  - Vectors (5)  **Movement**  - Trade and barter (6)  **Geographical**  - Villages locations (7) | 1, 2, 3, 6, 7 | 5 | 4, 5 | 1, 3, 6 | 2 |
| Barker et al. (2013) [20] | **Experimental**  - EIP duration variations with temperature (1)  **Environmental**  - Temperature (2)  - Land use (3)  **Demographic**  - Livestock (4)  - Vectors (5)  - Birds (6) | 2, 3, 4, 6 | 2, 5 | 1, 5 | 2, 3, 4, 6 |  |
| Gachohi et al. (2016) [6] | **Environmental**  - Rainfall (1)  **Epidemiological**  - Vaccination data (2)  - Outbreaks dates (3)  **Demographic**  - Livestock + age structure (4) | 1 | 1 | 1, 2, 3, 4 | 1 |  |
| Pedro et al. (2016) [32] | **Epidemiological**  - Prevalence (livestock) (1)  - Outbreaks years (2) |  | 1, 2 |  |  | 1, 2 |

**Table C - Continued**

| **Study** | **Type of data used** (numbers defined in this column are used in the following columns) | **Spatialized data** | **Time series data** | **Use of data** | | |
| --- | --- | --- | --- | --- | --- | --- |
|  |  |  |  | **Calibration** | **Input** | **Inference / model assessment** |
| Cavalerie et al. (2015) [33] | **Epidemiological**  - Outbreaks start (1)  - Duration of presence of the virus (2)  - Serological (animals) (3)  **Demographic**  - Vectors (4) |  | 3 | 1, 4 |  | 2, 3 |
| Bicout and Sabatier (2004) [34] | **Environmental**  - Rainfall (1)  **Epidemiological**  - Rate of decay of IgG antibodies positive individuals (livestock) (2)  **Demographic**  - Vectors (3) |  | 1, 3 | 1, 2, 3 |  |  |
| Tuncer et al. (2016) [35] | **Experimental**  - TCID50 (infectious virus), IgM and IgG antibodies in sheep (1)  **Epidemiological**  - Incidence (humans) (2) |  | 1, 2 |  |  | 1, 2 |
| Scoglio et al. (2016) [25] | **Demographic**  - Livestock (1)  - Vectors (2)  **Geographical**  - Farms locations (3) | 1, 3 |  | 2 | 1, 3 |  |

**Table C - Continued**

| **Study** | **Type of data used** (numbers defined in this column are used in the following columns) | **Spatialized data** | **Time series data** | **Use of data** | | |
| --- | --- | --- | --- | --- | --- | --- |
|  |  |  |  | **Calibration** | **Input** | **Inference / model assessment** |
| Xue et al. (2012) [19] | **Environmental**  - Temperature (1)  - Rainfall (2)  - Wind (3)  **Epidemiological**  - Incidence (livestock) (4)  - Incidence (humans) (5)  **Demographic**  - Livestock (6)  - Human (7)  **Movement**  - Trade (8)  - Commuting/return rates (humans) (9)  **Geographical**  - Distance between patch centers (10) | 3, 4, 5, 10 | (1, 2 : assumed),  4, 5 |  | 1, 2, 3, 6, 7, 8, 9, 10 | 4, 5 |
| Xue et al. (2013) [36] | **Environmental**  - Temperature (1)  - Rainfall (2)  **Demographic**  - Livestock (3)  **Movement**  - Aggregated movements (livestock) (4)  **Geographical**  - Distance between node centers (5) | 3, 5 | 1, 2 | 4 | 1, 2, 3, 5 |  |
| Beechler et al. (2015) [37] | **Epidemiological**  - Serological (animals) (1)  - BTB status (2) |  | 1 |  |  | 1, 2 |
| Sekamatte et al. (2019) [26] | **Demographic**  - Livestock (1)  **Geographical**  - Centroids (longitude and latitude) (2) | 1 ,2 |  |  | 1, 2 |  |

*EIP: extrinsic incubation period. BTB: bovine tuberculosis.*

**Table C - Continued**

| **Study** | **Type of data used** (numbers defined in this column are used in the following columns) | **Spatialized data** | **Time series data** | **Use of data** | | |
| --- | --- | --- | --- | --- | --- | --- |
|  |  |  |  | **Calibration** | **Input** | **Inference / model assessment** |
| EFSA AHAW Panel et al. (2020 - Model 1) [9] | **Environmental**  - Rainfall (1)  **Epidemiological**  - Serological (livestock) (2)  **Demographic**  - Livestock (3) |  | 1, 2 | 2 | 1, 3 |  |
| EFSA AHAW Panel et al. (2020 - Model 2) [9] | **Environmental**  - Temperature (1)  **Demographic**  - Livestock (2)  **Geographical**  - Farms locations (3) | 1, 2, 3 | 1 |  | 1, 2, 3 |  |
| Tennant et al. (2021) [28] | **Environmental**  - Vegetation (1)  **Epidemiological**  - Serological (livestock) (2)  **Demographic**  - Livestock (3)  **Movement**  - Trade (4) | 1, 2, 3 | 1, 2 | 4 | 1, 3 | 2 |
| Lo Iacono et al. (2018) [38] | **Experimental**  - Larvae and pupae mortality rate according to temperature (1)  **Environmental**  - Temperature (2)  - Water body surface (3)  **Epidemiological**  - Duration of interepidemic periods (4) |  | 2, 3 | 1 | 2, 3 | 4 |

**References**

1. Gaff H, Burgess C, Jackson J, Niu T, Papelis Y, Hartley D. Mathematical model to assess the relative effectiveness of Rift Valley fever countermeasures. International Journal of Artificial Life Research. 2011 Apr;2(2):1–18.

2. Adongo D, Fister KR, Gaff H, Hartley D. Optimal control applied to Rift Valley fever. Natural Resource Modeling. 2013 Aug 1;26(3):385–402.

3. Chamchod F, Cantrell RS, Cosner C, Hassan AN, Beier JC, Ruan S. A modeling approach to investigate epizootic outbreaks and enzootic maintenance of Rift Valley fever virus. Bull Math Biol. 2014 Aug;76(8):2052–72.

4. Chamchod F, Cosner C, Cantrell RS, Beier JC, Ruan S. Transmission dynamics of Rift Valley fever virus: effects of live and killed vaccines on epizootic outbreaks and enzootic maintenance. Front Microbiol. 2016;6.

5. Yang CX, Nie LF. Modelling the use of impulsive vaccination to control Rift Valley fever virus transmission. Advances in Difference Equations. 2016 May 18;2016(1):134.

6. Gachohi JM, Njenga MK, Kitala P, Bett B. Modelling vaccination strategies against Rift Valley fever in livestock in Kenya. PLoS Negl Trop Dis. 2016;10(12):e0005049.

7. McMahon BH, Manore CA, Hyman JM, LaBute MX, Fair JM. Coupling vector-host dynamics with weather geography and mitigation measures to model Rift Valley fever in Africa. Mathematical Modelling of Natural Phenomena. 2014;9(2):161–77.

8. Métras R, Edmunds WJ, Youssouffi C, Dommergues L, Fournié G, Camacho A, et al. Estimation of Rift Valley fever virus spillover to humans during the Mayotte 2018-2019 epidemic. Proc Natl Acad Sci U S A. 2020 29;117(39):24567–74.

9. EFSA AHAW Panel, Nielsen SS, Alvarez J, Bicout DJ, Calistri P, Depner K, et al. Rift Valley fever – assessment of effectiveness of surveillance and control measures in the EU. EFSA Journal. 2020;18(11):e06292.

10. Niu T, Gaff HD, Papelis YE, Hartley DM. An epidemiological model of Rift Valley fever with spatial dynamics. An GC, editor. Computational and Mathematical Methods in Medicine. 2012 Aug 13;2012:138757.

11. Xue L, Scoglio C. The network level reproduction number for infectious diseases with both vertical and horizontal transmission. Mathematical Biosciences. 2013 May 1;243(1):67–80.

12. Xue L, Scoglio C. Network-level reproduction number and extinction threshold for vector-borne diseases. Mathematical Biosciences and Engineering. 2015;12(3):565–84.

13. Wen B, Teng Z, Liu W. Threshold dynamics in a periodic three-patch Rift Valley fever virus transmission model. Complexity. 2019 Jan 9;2019:1–18.

14. Python Ndekou Tandong P, Ndiaye PI, Bah A, Dione D, Ndione JA. Coupling an agent-based model with a mathematical model of Rift Valley fever for studying the Impact of animal migrations on the Rift Valley fever transmission. In: Gervasi O, Murgante B, Misra S, Garau C, Blečić I, Taniar D, et al., editors. Computational Science and Its Applications – ICCSA 2020. Cham: Springer International Publishing; 2020. p. 471–85.

15. Gao D, Cosner C, Cantrell RS, Beier JC, Ruan S. Modeling the spatial spread of Rift Valley fever in Egypt. Bull Math Biol. 2013 Mar 1;75(3):523–42.

16. Mpeshe SC, Luboobi LS, Nkansah-Gyekye Y. Modeling the impact of climate change on the dynamics of Rift Valley fever. Comput Math Methods Med. 2014;2014:627586.

17. Xiao Y, Beier JC, Cantrell RS, Cosner C, DeAngelis DL, Ruan S. Modelling the effects of seasonality and socioeconomic impact on the transmission of Rift Valley fever virus. PLoS Negl Trop Dis. 2015 Jan 8;9(1):e3388.

18. Sumaye R, Jansen F, Berkvens D, De Baets B, Geubels E, Thiry E, et al. Rift Valley fever: An open-source transmission dynamics simulation model. PLoS ONE. 2019 Jan 9;14(1):e0209929.

19. Xue L, Scott HM, Cohnstaedt LW, Scoglio C. A network-based meta-population approach to model Rift Valley fever epidemics. J Theor Biol. 2012 Aug;306:129–44.

20. Barker CM, Niu T, Reisen WK, Hartley DM. Data-driven modeling to assess receptivity for Rift Valley fever virus. PLoS Neglected Tropical Diseases. 2013 Nov 14;7(11):e2515.

21. Fischer EAJ, Boender GJ, Nodelijk G, de Koeijer AA, van Roermund HJW. The transmission potential of Rift Valley fever virus among livestock in the Netherlands: a modelling study. Vet Res. 2013 Jul 22;44:58.

22. Nicolas G, Chevalier V, Tantely LM, Fontenille D, Durand B. A spatially explicit metapopulation model and cattle trade analysis suggests key determinants for the recurrent circulation of Rift Valley fever virus in a pilot area of Madagascar highlands. PLoS Negl Trop Dis. 2014 Dec 4;8(12):e3346.

23. Leedale J, Jones AE, Caminade C, Morse AP. A dynamic, climate-driven model of Rift Valley fever. Geospat Health. 2016 Mar 31;11(1 Suppl):394.

24. Taylor D, Hagenlocher M, Jones AE, Kienberger S, Leedale J, Morse AP. Environmental change and Rift Valley fever in eastern Africa: projecting beyond HEALTHY FUTURES. Geospat Health. 2016 Mar 31;11(1 Suppl):387.

25. Scoglio CM, Bosca C, Riad MH, Sahneh FD, Britch SC, Cohnstaedt LW, et al. Biologically informed individual-based network model for Rift Valley fever in the US and evaluation of mitigation strategies. PLoS ONE. 2016 Sep 23;11(9):e0162759.

26. Sekamatte M, Riad MH, Tekleghiorghis T, Linthicum KJ, Britch SC, Richt JA, et al. Individual-based network model for Rift Valley fever in Kabale District, Uganda. PLoS ONE. 2019 Mar 5;14(3):e0202721.

27. Cecilia H, Métras R, Fall AG, Lo MM, Lancelot R, Ezanno P. It’s risky to wander in September: Modelling the epidemic potential of Rift Valley fever in a Sahelian setting. Epidemics. 2020 Dec 1;33:2020.02.25.20027821.

28. Tennant WSD, Cardinale E, Cêtre-Sossah C, Moutroifi Y, Le Godais G, Colombi D, et al. Modelling the persistence and control of Rift Valley fever virus in a spatially heterogeneous landscape. Nat Commun. 2021 Sep 22;12(1):5593.

29. Gil H, Qualls WA, Cosner C, DeAngelis DL, Hassan A, Gad AM, et al. A model for the coupling of the Greater Bairam and local environmental factors in promoting Rift Valley fever epizootics in Egypt. Public Health. 2016 Jan 1;130:64–71.

30. Métras R, Fournié G, Dommergues L, Camacho A, Cavalerie L, Mérot P, et al. Drivers for Rift Valley fever emergence in Mayotte: a Bayesian modelling approach. PLoS Negl Trop Dis. 2017 Jul 21;11(7):e0005767.

31. Durand B, Lo Modou M, Tran A, Ba A, Sow B, Belkhiria J, et al. Rift Valley fever in northern Senegal: a modelling approach to analyse the processes underlying virus circulation recurrence. PLoS Negl Trop Dis. 2020 Jun 1;14(6).

32. Pedro SA, Abelman S, Tonnang HEZ. Predicting Rift Valley fever inter-epidemic activities and outbreak patterns: insights from a stochastic host-vector model. PLoS Neglected Tropical Diseases. 2016 Dec 21;10(12):e0005167.

33. Cavalerie L, Charron MVP, Ezanno P, Dommergues L, Zumbo B, Cardinale E. A stochastic model to study Rift Valley fever persistence with different seasonal patterns of vector abundance: new insights on the endemicity in the tropical island of Mayotte. PLoS ONE. 2015 Jul 6;10(7):e0130838.

34. Bicout DJ, Sabatier P. Mapping Rift Valley fever vectors and prevalence using rainfall variations. Vector Borne Zoonotic Dis. 2004;4(1):33–42.

35. Tuncer N, Gulbudak H, Cannataro VL, Martcheva M. Structural and practical identifiability issues of immuno-epidemiological vector–host models with application to Rift Valley fever. Bulletin of Mathematical Biology. 2016 Sep;78(9):1796–827.

36. Xue L, Cohnstaedt LW, Scott HM, Scoglio C. A hierarchical network approach for modeling Rift Valley fever epidemics with applications in North America. PLoS ONE. 2013 May 7;8(5):e62049.

37. Beechler BR, Manore CA, Reininghaus B, O’Neal D, Gorsich EE, Ezenwa VO, et al. Enemies and turncoats: bovine tuberculosis exposes pathogenic potential of Rift Valley fever virus in a common host, African buffalo *(Syncerus caffer*). Proceedings of the Royal Society B: Biological Sciences. 2015 Apr 22;282(1805):20142942.

38. Lo Iacono G, Cunningham AA, Bett B, Grace D, Redding DW, Wood JLN. Environmental limits of Rift Valley fever revealed using ecoepidemiological mechanistic models. Proc Natl Acad Sci USA. 2018 31;115(31):E7448–56.
