# Supplementary material for: Mechanistic models of Rift Valley fever virus transmission: A systematic review
Source: PLoS Negl Trop Dis. 2022 Nov 18;16(11):e0010339. doi: 10.1371/journal.pntd.0010339 (PMC9718419; doi:10.1371/journal.pntd.0010339)
Supplement: Attachment — Submitted filename: Response to reviewers.docx [file pntd.0010339.s002.docx]

Dear Mr. Drouin,

Thank you very much for submitting your manuscript "Mechanistic models of Rift Valley fever virus transmission dynamics: A systematic review" for consideration at PLOS Neglected Tropical Diseases. As with all papers reviewed by the journal, your manuscript was reviewed by members of the editorial board and by several independent reviewers. In light of the reviews (below this email), we would like to invite the resubmission of a significantly-revised version that takes into account my and the reviewers' comments.

**General comment: I found this to be an interesting review of the various models of RVF, but as I am not a modeler, I was a little concerned as I could not tell which of the models cited were good and which were not, i.e., simply stating that “Eleven models (22%) incorporated the influence…” (line 496) doesn’t indicate how well these models did. One of the models incorporated ticks (see my comment 5 below) and I am not certain about many of the assumptions made on potential vectors. I also made a few minor editorial comments.**

*Please note that lines numbers refers to the final version, without tracked changes.*

We would like to thank the editor for the careful reading and the positive comments. In our opinion, it is quite difficult to simply qualify a model as ‘good’ or not, or to compare the way they tested hypotheses or incorporated new mechanisms in an impartial way. Only models using field data to validate their results can be properly evaluated, but this was only the case for 12 models (Lines 464-465, Table C in S1). Due to the lack of epidemiological data, most studies presented in this review are rather a way to explore model behaviour under certain hypotheses, some well established, other more exploratory, to highlight possible patterns needing further field exploration.

We have enriched our conclusion to express how we think this diversity of models should be used, lines 624-630 : “This increasing number of models could also reflect a growing trust in mechanistic models in the field of infectious disease epidemiology [158, 159]. When it comes to decision-making for disease management, we agree with previous work showing that combining models is the most sensible approach rather than attempting to find the best model [160, 161]. Indeed, the diversity of models’ structure and hypotheses is a richness, which can be used to highlight actions that are robust to model uncertainty, but also identify key differences needing clarification through additional field exploration [160, 161].”

We respond to each of specific comments in the following section.

**Specific comments**:

**1.  Line 44: Yes, RVFV is primarily associated with Aedes and Culex mosquitoes, but numerous species of other mosquito genera have been found to be naturally infected in the field and shown to be competent vectors of RVFV in the laboratory. Depending on the situation, these species may play a critical role.**

We agree with this comment and modified the sentence in lines 43-45: ‘This disease is one of the World Health Organization priorities, and is caused by a virus which is transmitted by mosquitoes (mainly of *Aedes* and *Culex* spp. genera) mosquitoes, but also by direct contact from livestock to humans.’ We also changed lines 57-59: ‘Transmission of Rift Valley fever virus (RVFV) often involves *Aedes* and *Culex* spp. mosquitoes [2], some of which are present in Europe and North America [3–10]. Other genera, such as *Anopheles* and *Mansonia*, are also possible vectors [11,12].’

**2.  Line 58: All of the papers cited deal with European mosquitoes, but the sentence also says, “North America.” Why not also include “Turell MJ, Dohm DJ, Mores CN, Terracina L, Wallette DL Jr, Hribar LJ, et al. Potential for North American mosquitoes (Diptera: Culicidae) to transmit Rift Valley fever virus. J Am Mosq Control Assoc. 2008;24:502-7?”**

We added the suggested reference, along with three others (line 58):
- Hartman DA, Bergren NA, Kondash T, Schlatmann W, Webb CT, Kading RC. Susceptibility and barriers to infection of Colorado mosquitoes with Rift Valley fever virus. PLoS Negl Trop Dis. 2021 Oct 25;15(10):e0009837 (ref number 9)
- Turell MJ, Dohm DJ, Mores CN, Terracina L, Wallette DL, Hribar LJ, et al. Potential for North American Mosquitoes to Transmit Rift Valley Fever Virus. moco. 2008 Dec;24(4):502–7. (ref number 7)
- Turell MJ, Wilson WC, Bennett KE. Potential for North American Mosquitoes (Diptera: Culicidae) to Transmit Rift Valley Fever Virus. Journal of Medical Entomology. 2010 Sep 1;47(5):884–9. (ref number 8)
- Iranpour M, Turell MJ, Lindsay LR. Potential for Canadian mosquitoes to transmit Rift Valley fever virus. J Am Mosq Control Assoc. 2011 Dec;27(4):363–9. (ref number 10)

**3.  Line 276: What do you mean by a vertical transmission rate of 10%? I assume that you mean than 10% of the orally infected mosquitoes produced at least 1 infected progeny and not that 10% of the progeny of the infected mosquitoes were infected. Given that vertical transmission is very rare to the first ovarian cycle progeny, and most of the infected mosquitoes won’t survive to produce a second cycle, how are you calculating this? What about stabilized infections, i.e., where in a very small percent of the mosquitoes, virtually 100% of their progeny, including first ovarian cycle progeny, are infected, and this persists for many, many generations. This is likely what allows RVFV to remain enzootic in a particular region.**

In the deterministic model described by Pedro et al. (2016), the vertical transmission rate corresponds to the proportion of infected eggs produced by an infected female. The value of this rate was not computed by us but simply reported from the modeling paper. Pedro et al. (2016) cited Romoser et al. 2011 “*Rift Valley fever virus-infected mosquito ova and associated pathology: possible implications for endemic maintenance*” to justify this value of 10%. However, Romoser et al. reported 8.6% of females having a single infected egg, which indeed has a very different meaning. In addition, Pedro et al. found that the substantial effect on $R_{0}$ was observed with 20% vertical transmission instead of 10% as we wrote before.

We changed the sentences in lines 278-282 to highlight that this rate seems to be unrealistically high:
‘Pedro et al. (2016) [72] (theoretical) estimated a linear and significant effect of vertical transmission on R0 and vector eradication effort although this effect became substantial only when vertical transmission rate was above 20% (percentage of infected mosquitoes’s progreny which are infected). However, such a high rate seems to be unrealistic given experimental observations [110].”

Regarding the stabilized infection (mentioned in Romoser et al.,2011), none of the included models took this mechanism into account. We added in conclusion of this paragraph in lines 288-296:

‘The inconsistent conclusions from those models might indicate a spatially and temporally heterogeneous role of vertical transmission in RVFV maintenance. Moreover, most models have considered a uniform vertical transmission rate. However, it is more likely that the percentage of infected progeny may vary depending on individuals. For instance, it has been evidenced in *Aedes dorsalis* the possible existence of 'stabilized infections' for the California encephalitis virus [108], i.e., a very small percent of mosquitoes are able to infect virtually 100% of their progeny, so that infection in mosquitoes is able to persist over several generations. Models could be used to explore that scenario, as the same mechanism has been suggested for RVFV [110].’

**4.  Line 277: Why now use “transovarial” instead of vertical transmission. Yes, transovarial is likely to be the most important route of vertical transmission, but there is also transovum transmission. I would only use “vertical transmission.”**

We changed ‘transovarial’ to ‘vertical’ throughout the paper.

**5. Line 295: Yes, Pedro et al. (2017) looked at what might happen if ticks could transmit RVFV. However, the last sentence of their abstract is, “These findings suggest that if ticks are capable of transmitting the virus, they may be contributing to disease outbreaks and endemicity.” Has anyone ever shown that an orally exposed tick, of any species, is able to transmit RVFV? If not, then why not include mites, lice, and various biting flies?**

To our knowledge, there is no evidence of transmission of RVFV from orally exposed ticks. Linthicum et al. (1989) demonstrated that inoculated *Hyalomma truncatum* transmitted the virus to hamsters, but not the orally exposed ticks. This study is cited by Pedro et al. (2017) but is indeed not conclusive on ticks being possible vectors. We added the sentence ‘It should be noted, however, that there is currently no evidence of the ability of ticks to biologically transmit the virus [112]’ (Lines 315-316) to be more explicit on the strong hypothesis made by the authors.

**6.  Line 336-338: Here the authors are stating that if “even with R0 < 1, RVF was likely to become endemic when live vaccines were implemented.” Do they mean that if a killed vaccine was used that RVF would NOT become enzootic? Note, as RVF is primarily a disease of animals, then shouldn’t it be enzootic, rather than endemic?**

In situations with $R_{0}$ < 1, RVF is indeed not expected to become enzootic, and this is still the case with the used of killed vaccines. What is emphasized here is that according to Chamchod et al. (2016) RVFV could become enzootic even with R0 < 1 when using live vaccine due to the reversion of virulence. Endemic was the word used by Chamchod et al., but we changed it to enzootic in our text, as the model does not include humans. We clarified the sentence: ‘Chamchod et al. (2016) [105] explored differences between the use of live and killed vaccines, and showed that due to the associated reversion of virulence, the use of live vaccines could render RVFV enzootic in situations where $R_{0}$ is initially below one.’ (Lines 353-356)

**7.  Line 340: Yes, everyone refers to larval developmental sites as “breeding” sites, but “breeding” has a sexual connotation, and mosquitoes do not “breed” in these locations. It would be more accurate to refer to them as “larval developmental sites.”**

Amended in line 358.

**8.  Line 355-357: Was it the “movement restriction between Grande Comore and other islands of the archipelago led to a more severe and delayed outbreak” or were other factors also involved that actually caused the more severe outbreak?**

This sentence refers to results of modelling scenarios, in which the authors were able to change only one parameter and thus to link observed results to the control measure tested. We modified the sentence to be clearer: ‘In Comoros, scenarios of movement restriction between Grande Comore and other islands of the archipelago delayed the outbreak to a more suitable season, making it more severe overall [87]. By contrast, within-island control appeared to be more effective.’ (Lines 373-375).

**9.  Lines 453-454: Yes, “livestock” does appear to be a single vertebrate host, but what if the area had cattle, goats, and sheep? These would all be considered livestock, but a certainly not a single vertebrate host.**

We modified the sentence to precise that these models included a single host *species,* not a ‘single host’, as the absence of distinction between species is what we wanted to highlight: ‘Most models (34/49) included a single vertebrate host species, most of the time broadly labeled as livestock without making distinction between species (25/34, Table 1).’ (Lines 477-478)

**10.  Lines 458-459: Yes, as incompetent vertebrate hosts, birds might interfere with RVFV transmission. However, many of the mosquitoes, particularly the Aedes, that are involved in RVFV transmission preferentially feed on mammals. Therefore, whether birds were present or not would make little difference. This is unlike the situation with cattle as an alternative host for Anopheles mosquitoes and malaria, where the presence of cattle near a home may provide an attractive alternative host that was incompetent for human malaria and thus reduces malaria in the area.**

In the reference including birds as dead-end hosts (Barker et al., 2013), two mosquitoes species were considered: *Aedes melanimon* and *Culex tarsalis,* both being allowed to feed on birds in the model. *Cx. melanimon* is here presented as an opportunistic feeder, with a greater number of blood meals taken on avian species compared to mammal species, depending on the time of the year (this is based on two studies conducted in California, i.e., in the same area where the model is set). Considering *Ae. melanimon,* Barker and collaborators indicated that this species preferentially feed on mammals, and since mammal hosts are predominant in most pixels of their model settings, they remain the main blood meal source for mosquitoes. We added the vectors names in the sentence for clarity (Line 484).

**11.  Line 486: Why would humans be a dead-end host? Viremias in humans are extremely high, often more than 8 logs, i.e., higher than often observed in adult cattle, goats, or sheep. This is unlike viruses such as West Nile virus and Japanese encephalitis virus in which humans produce such a low viremia that they are indeed dean-end hosts.**

We clarified (Lines 507-515): “Among eleven models including a human compartment (Table 1), nine considered livestock-to-human transmission by direct route (without vector) and ten models considered mosquito-to-human transmission. From these ten models, three [69,91,100] considered human-to-mosquito transmission. This is in line with human being often considered as dead-end hosts in the literature [18,137]. However, some data, while scarce, suggest they could develop a high viremia [138,139], which would be sufficient to infect mosquitoes. Considering the difficulty to obtain direct observations on that matter, it would seem relevant for future models to evaluate whether human-to-mosquito transmission is necessary to explain observed transmission dynamics.’

**12.  Line 491: Why would Ae. vexans be the most common in these models? The very few studies on the ability of Ae. vexans orally exposed to RVFV to transmit RVFV indicate that there is a very wide range in vector competence for this species, ranging from <1% in the northern U.S., 5% in Europe, to about 20% in the southeastern U.S. I know of no studies that have determined if African strains of Ae. vexans are competent vectors. Note, isolation of virus from a field-collected mosquito does NOT mean that it is capable of transmitting that virus. Various arboviruses, including RVFV, have been detected in mosquito species that have been shown to be incompetent and thus not vectors.**

*Ae. vexans* is indeed the most common vector among studies specifying the vector species they are modeling, but there are only 5/43 models including this species explicitly. We fully agree with the geographic variability of the competence of *Ae. vexans* for the RVFV, and we showed in a recent meta-analysis that *Ae. vexans* could be considered as a moderately competent vector, despite the large confidence intervals (Drouin A, Chevalier V, Durand B, Balenghien T. Vector Competence of Mediterranean Mosquitoes for Rift Valley Fever Virus: A Meta-Analysis. Pathogens. 2022 May;11(5):503.). In addition, we included in this work a study conducted on Senegalese wild-caught *Ae. vexans,* which demonstrated the vector competence of this species for the RVFV (infection, dissemination and transmission) for at least one viral strain. (Ndiaye EH, Fall G, Gaye A, Bob NS, Talla C, Diagne CT, et al. Vector competence of *Aedes vexans* (Meigen), *Culex poicilipes* (Theobald) and *Cx. quinquefasciatus* Say from Senegal for West and East African lineages of Rift Valley fever virus. Parasites & Vectors. 2016 Feb 20;9(1):94:1-94:9.).

**13.  Lines 504-506: yes, these models may have taken this into account, but the effect differs for different vectors, so how did they do that?**

Authors did not systematically consider differences of these mechanisms between species. We added a sentence to highlight authors who did: ‘Among them, Fischer et al. (2013), Lo Iacono et al. (2018), Mpeshe et al. (2014) and Durand et al. (2020) [69,88–90] considered differences between *Culex* and *Aedes* mosquitoes for EIP and/or biting rate.’(Lines 535-537)

We also added in conclusion of the paragraph (lines 545-548): “Overall, and due to the lack of data, modelling the impact of abiotic factors on the life cycle and competence of mosquitoes often relied on using data from different genus or species than those under study. In such cases, authors considered this choice preferable to a constant parameter or an arbitrary mathematical function.”

**14.  Lines 539-589: This is not as simple as it appears. For example, if 1 in 50 of the sheep in a flock is infected, most models would assume that 1 in 50 mosquitoes feeding on an animal in that flock would be exposed to virus. That is not correct. RVFV-infected animals are more attractive to a blood-seeking mosquito than their uninfected siblings, so greater than 1 in 50 would have been exposed to virus. Similarly, many mosquitoes may be dislodged before they obtain a blood meal, but mosquitoes are able to obtain a blood meal more rapidly from a RVFV-infected animal than from an uninfected member of the same species. Again, the successful feeding rate on RVFV-infected animals would be higher than the percent of animals actually infected. Have either of these been incorporated in the models?**

This section on force of infection (which has now been moved to a text box) intended to explain the underlying assumptions found in RVFV models and most vector-borne diseases models that we know of. Indeed most models consider a homogeneous mixing hypothesis, which is convenient but does not necessarily reflect what happens in real-life settings. We added a sentence to highlight that, lines 549-553:

‘In reviewed models, the only source of variability in the feeding behavior of vectors was the inclusion of trophic preference for one host species over the others [84,88,89,96]. However, studies have suggested that the infected or uninfected status of the host might also play a role, for different pathogens [143, 144], including for RVFV [145, 146]. Future models could incorporate this mechanism to test its epidemiological importance.’

**15.  References: Please ensure that all references are formatted correctly.**

**a. In manuscript titles, only the first word and all proper nouns should be capitalized. See references 1, 14 and others where “rift valley fever.” should have been “Rift Valley fever.” See also references 7, 8, 10, and others where each word is capitalized, even if not a proper noun. Please check all of the references.**

Done.

**b. See references 41 and 46. Is it PLOS ONE or PLoS ONE?**

All ‘PLOS’ occurrences have been changed to ‘PLoS’

**c. What does the “Larchmt N” mean in reference 54?**

This was an error and it has been erased.

**d. Why do you list the editor for some of the PLoS journals?**

This was an error and it has been corrected.

**e. Please go through all of the references and ensure that they are properly formatted.**
Note that reference 149 has just been accepted in *Plos Computational Biology* so it will have a proper citation by the time our paper is published.

**Minor comments**:

**16.  Line 44: Both “Aedes” and “Culex” should be in italics.**

Done. We checked every occurrences of these words.

**17.  Line 91: RVFV was established on line 57 and should be used here. See also lines 278, 290, 376, 466 etc.**

Done. We checked every occurrences of these words and replaced it.

**18.  Line 151, Figure 1: The first block indicates that 372 records were identified (236 + 136). The next block indicates that 123 of these were removed prior to screening. So why weren’t 249, instead of 248 screened? In all of the other reductions, the numbers do match?**

We checked every numbers of figure 1 and corrected the typo: 124 records were removed prior screening, not 123.

**19.  Line 169: Shouldn’t “4 cases…” be “four cases…” See also lines 367, 391-394, 421, etc. I am fine with using a numeral within a parentheses, but you need to be consistent outside of a parentheses.**

We modified the text to be consistent using letters writing for numbers from one to nine. We however tried to keep some homogeneity between numeral or written figures within sentences, when necessary.

**20.  Line 342: Is it really necessary, or even accurate, to present lifetime to a hundredth of a day? Why not simply state, “under 8.7 days…”**

We changed to 8.7 days.

**21.  Line 448: Why is 4/10 39% instead of 40%?**

We changed 39% to 40%.

**22.  Line 480: Is it RVF infection or RVFV infection? This occurs throughout the manuscript. Mosquitoes do not transmit RVF, they transmit the virus that causes RVF.**

We checked every occurrences of ‘RVF’ and changed it to ‘RVFV’ when necessary.

**23.  Lines 499-500: Shouldn’t “on the lifespan of adult vectors lifespan” be “on the lifespan of adult vectors?”**

We corrected the mistake.

**24.  Line 530: What is a SIR-type model? I assume that the authors are referring to a susceptible-infected-recovered model, but don’t know if the reader will get that. Also, in Table 1, is it “sensible” or “susceptible?” I think that susceptible is more accurate.**

We added the full letter model name in the sentence (now the first sentence of Box 1), and we corrected the mistake in the Table 1 legend.

**Reviewer #1: Editorial recommendations**:

**- ms is very readable and accessible; authors do well bringing the reader into their view and objectives; good balance of technical and narrative content**

We thank the Reviewer #1 for the positive comments.

**- make sure each "e.g." and "i.e." is followed by a comma**

Done.

**- use serial commas (i.e., make sure there is a comma before the "and" or the "or" in a list of three or more items in text, such as line 118 or line 398)**

All occurrences of ‘and’ and ‘or’ have been checked, and serial comma added when necessary.

**- check that parenthetical statements are properly closed with a ")" ... e.g., in line 76. Overall, I would go through the whole ms to minimize use of parenthetical statements by rewording. However, the way you use them in for example lines 125-129 makes sense and works well.**

We checked the whole manuscript and tried to erase parenthetical statements where this was not necessary.

**- check for correct grammar with the word "data" which is a plural term - I think there was only one place (line 429) but check throughout**

We checked all occurrences of this word and used plural when necessary.

**- be consistent with either numbering or using author/date in-text citations (e.g., in-text citations throughout the paragraph lines 365-376 are inconsistent with journal style)(on the other hand, the way you combine them in lines 159-171 makes sense and works well)**

We checked the whole manuscript and corrected in-text citations inconsistency when necessary.

**- check that "R" (as used in Ro, etc.) is italicized throughout**

We checked the whole manuscript and corrected it when necessary.

**- lines 93-94: suggest rewording "To achieve this, we categorized models as either theoretical or applied, and explored these categories throughout the paper to identify what they have in common and how they differ."**

We integrated the suggested rewording in text (now lines 96-68).

**- suggest find a way to make the count in line 31 and line 148 match (I understand it was 48 papers but 49 studies because one paper had two studies)**

Done.

**- lines 118-120: suggest rewording to "Records selected in the first and second step went to a full text screening of the corresponding report, using a combination of the first set of exclusion criteria along with these additional exclusion criteria:"**

We integrated the suggested rewording in text (now lines 121-123).

**- line 244 should be four categories not five?**

We modified the sentence for more clarity: ‘The main output of a model, holding the key message of the studies, could pertain to one of four main categories:’ (Line 243)

**- lines 482-485: suggest rewording for clarity ... do you mean "Among 11 models including a human compartment (Table 1), three [42,64,73] considered human-to-mosquito transmission, 10 considered mosquito-to-human transmission, and one [67] considered livestock-to-human transmission."**

We clarified, lines 507-515: ‘Among eleven models including a human compartment (Table 1), nine considered livestock-to-human transmission by direct route (without vector) and ten models considered mosquito-to-human transmission. From these ten models, three [69,91,100] considered human-to-mosquito transmission. This is in line with human being often considered as dead-end hosts in the literature [18,137]. However, some data, while scarce, suggest they could develop a high viremia [138,139], which would be sufficient to infect mosquitoes. Considering the difficulty to obtain direct observations on that matter, it would seem relevant for future models to evaluate whether human-to-mosquito transmission is necessary to explain observed transmission dynamics.’

**Reviewer #1: First I want to thank you for a very well written, timely, and informative paper.
This manuscript synthesizes 48 Rift Valley fever virus modeling papers (49 studies) using very creative methods of analysis and communication of findings.**

**The figures are very well executed and valuable, and greatly enhance the text descriptions and tabulated analysis.**

**The ‘reading grid’ structured analysis of the papers makes a lot of sense and is a very defendable method for bringing together input from multiple co-authors.
The objective of the study is to summarize a state-of-the-science which also includes a gap analysis to guide future research.**

**The methodical, stepwise discussion of how key features of mechanistic disease models are handled across the studies will be very useful not only to guide future project designs, but also is very instructive and accessible for early career modelers.**

We thank the Reviewer #1 for the careful reading and the positive comments.

**I have a few recommendations to improve the ms for publication:**

**Lines 75-76 … can you elaborate on why phenomenological should be used instead of statistical?**

We have modified the text (Lines 76-80): “Phenomenological models, be they mathematical or statistical, aim at extracting patterns and information from data, with no focus on underlying mechanisms responsible for such observed patterns [29]. By contrast, mechanistic (sometimes called dynamical) models explicitly include processes governing the system of interest [29]. Consequently, mechanistic models can adapt to data-scarce...”

**Lines 93-95 … should you mention the “gray” category here?**

Done: ‘we categorized models on a spectrum from theoretical to applied (the middle-ground category being called ‘grey’)’. (Line 96)

**Lines 98-99 … Force of Infection section (lines 524-621) … this section is very useful and interesting but possibly could be in the SI? I only say this because the intro paragraph (525-529) could almost skip straight to the “results” paragraph (starting 628) where you bring the narrative back to the papers you analyzed. As a reader, it left me a little lost until I understood what you were doing with the 524-621 section (i.e., setting the basis for your analysis and comment). Moving 524-621 to SI could keep the reader focused on your findings. Also, given that you devoted so much to the FOI issue, I expected more exploration and impact of this topic in the Conclusions.**

We chose to move the didactic content of lines 524-621 into a text box so that it’s more clearly separated from the results of the systematic review. We also added in the conclusion (Lines 618-623): “Indeed, as it reflects assumptions made on the contact rate between host and vector populations, this choice crucially influences model predictions and therefore cannot be made lightly. This systematic review showed that, as was the case for West Nile virus [150], models of RVFV transmission dynamics make very distinct assumptions which render their results not directly comparable. We detailed them didactically, hoping to guide future models focusing on vector-borne transmission”.

The Results section is now more brief and focuses on what is found in the models (lines 565-607):

‘We chose to focus on the diversity of functional forms (FFs) used in RVF models for the force of infection related to vector-borne transmission. This was applied only to models explicitly including a vector compartment. Among those, a majority (29/43) did not justify their choice of FF, even though the force of infection, as a disease transmission term, encapsulates authors’ assumption on the host-vector interactions, and therefore influences their predictions (Figure 5, [150]).’

Six FFs were found in reviewed models (Table 1, Figure 5). We detail them in Box 1. Thirteen models used a reservoir frequency-dependent FF (Eq. 1 in Box 1, Table 1, Figure 5). Eight models used a mass action FF (Eq. 2 in Box 1, Table 1, Figure 5). Twelve models used an infectious frequency-dependent FF (Eq. 3 in Box 1, Table 1, Figure 5). Ten models used alternative FFs, which all intended to avoid the shortcomings of other FFs by introducing parameters to constrain the contact rate between host and vector populations (Eq. 4-6 in Box. 1, called Hybrid1 (n=8), Hybrid2 (n=1), and Hybrid3 (n=1) in Table 1 and Figure 5).

In 82% of cases (14/17), a model used the same functional form (FF) for its force of infection as its parent model (Figure 2). In 9/14 cases, the parent model did not justify the choice of FF used, and no further justification was provided in the subsequent model in 7/9 cases. In 3/17 cases, the FF was changed compared to the parent model, which induced a justification in 2/3 cases. In addition, in 3 cases, the representation of vectors was implicit in a model and its parent model, therefore preventing the classification of the force of infection into any FF.

Several review papers on various epidemiological models concluded that the choice of a functional form for the force of infection could greatly affect model behavior. Begon et al. (2002), Hoch et al. (2018) and McCallum et al. (2001) [151,153,154] focused on non-vectorial transmission. Hopkins et al. (2020) [155] focused on parasite transmission, which could be through a vector, but did not include possible variations in frequency-dependent functions. Wonham et al. (2006) [150] focused on FFs used to model vectorial transmission of West Nile virus and also noticed an important diversity. In 2001, McCallum et al. were already recommending to ‘explicitly state and justify the form of transmission used" as well as "evaluate several alternative models of transmission, if possible" [154]. Contact structures between host and vector populations are hard to observe in natural conditions. This should be an additional incentive for modelers to explicitly state the reasoning behind their choice of functional form, which can be motivated by the context of their case study (e.g., expected host and vector densities, mixing between the populations). A comparison of the FF listed presently would be useful. The conclusions might vary depending on whether this is done through theoretical scenarios, keeping all other parameters equal, or by fitting different models to a common empirical dataset. The latter might not be able to discriminate between FF to select the best-performing one, because of underlying correlations between parameters.”

Other parts of the paragraph have been moved to Box 1 entitled **‘**Diversity of assumptions and functional forms for the force of infection in models of RVFV transmission dynamics’.

**Lines 247-263 (and possibly other sections, such as line 448) … the tallies you provide for the number of studies that attain various criteria throughout this paragraph are not identifiable by the reader to the specific studies. This is in contrast to other such tallies elsewhere in the ms that reference for example the Tables where the reader can translate each tally into the specific studies. You do reference Fig 3 but I don’t see a way to get to the specific studies tallied in 247-263 via this figure. This could be resolved by an additional table in SI that accompanies Fig. 3. Or maybe the existing tables have the relevant info and just need to be referenced in text?**

The main models objectives and output are detailed in Table 1: we now refer to Table 1 in line 254.

**Line 387 … could you list the countries?**

Countries have been listed in lines 406-407.

**Line 423 … is spatialized the same as data that are organically spatial? Or does it mean the data were fitted by the modeler to a spatial context?**

‘Spatialized data’ refers to datasets in which data are linked to a spatial information (measures in at least two distinct locations).

We clarified lines 443-445: “Only 44% of all datasets used by models incorporated a spatial dimension (measures in at least two distinct locations), and 45% a time dimension (measures for at least two different time points)”

**Line 428 … could you describe what the specific data gaps were in those cited studies?**

We added in the text (Lines 449-452): ‘Specifically, such gaps included the lack of fine-scale geographical metadata, preventing the study of within-country variation ; the need for long-term studies in both endemic and non-endemic countries, to evaluate a possible increase in RVFV activity and exposure ; and studies considering wildlife, livestock, and human concurrently, using standardized reporting and uniform case definitions [22,23].’

**Line 468 … could add “Britch SC, Binepal YS, Ruder MG, Kariithi HM, Linthicum KJ, Anyamba A, et al. Rift Valley fever risk map model and seroprevalence in selected wild ungulates and camels from Kenya. PLoS One. 2013;8: e66626”**

Done.

**Line 480 … should be RVFV infection (not RVF infection)**

We corrected it.

**Lines 485-487 … recommend citing Njenga et al. 2009 because humans can develop viremia sufficient to infect mosquitoes (Njenga MK, Paweska J, Wanjala R, Rao CY, Weiner M, Omballa V, et al. Using a field quantitative real-time PCR test to rapidly identify highly viremic Rift Valley fever cases. J Clin Microbiol. 2009;47: 1166–1171). Also Rolin et al. 2013 is a good reference for the idea of humans as dead end hosts (Rolin AI, Berrang-Ford L, Kulkarni MA. The risk of Rift Valley fever virus introduction and establishment in the United States and European Union. Emerg Microbes Infect. 2013;2: e81). We have recently submitted a review paper on the risk of spread of RVFV via infected humans - unfortunately still in review but will share with you if you are interested.**

We added the suggested references (Lines 511-512), and we are very interested into reading your review once published.

**Lines 512-513 … the NDVI basis for modeling RVFV risk was pioneered by Anyamba and Linthicum … I realize their models may have not met your criteria for inclusion in this ms but the NDVI technique for understanding RVFV transmission risk should be cited to them (Linthicum KJ, Anyamba A, Tucker CJ, Kelley PW, Myers MF, Peters CJ. Climate and satellite indicators to forecast Rift Valley fever epidemics in Kenya. Science. 1999;285: 397–400; Anyamba A, Linthicum KJ, Mahoney R, Tucker CJ, Kelley PW. Mapping potential risk of Rift Valley fever outbreaks in African savannas using vegetation index time series data. Photogramm Eng Remote Sens. 2002;68: 137–145.) … in particular because the predictions of the Rift Valley Fever Monitor (**[**https://www.ars.usda.gov/sout**](https://www.ars.usda.gov/southeast-area/gainesville-fl/center-for-medical-agricultural-and-veterinary-entomology/docs/rvf_monthlyupdates/)**) guided mass vaccination and prevention of an epizootic (Linthicum KJ, Britch SC, Anyamba A. Rift Valley fever: An
emerging mosquito-borne disease. Annu Rev Entomol. 2016;61: 395–415; Anyamba A, Chretien J-P, Britch SC, Soebiyanto RP, Small JL, Jepsen R, et al. Global Disease Outbreaks Associated with the 2015-2016 El Niño Event. Sci Rep. 2019;9: 1930.**

We added a sentence at the end of the paragraph, with the two suggested references: ‘This type of data have been used previously to map RVFV transmission risk [141,142].’ (Line 544)

**Lines 659-660: yes absolutely … this is a very timely and operationally relevant conclusion and direction for future research. Movement (legal, illegal) of infected livestock among regions in RVFV endemic areas is becoming more and more important. Increasing instances of interepizootic transmission of RVFV to humans throughout the African continent and the Arabian Peninsula driven by movement of infected livestock (Eisa et al. 1980, Meegan and Bailey 1989, Balkhy and Memish 2003, LaBeaud et al. 2008, Mohamed et al. 2014, Napp et al. 2018, Tigoi et al. 2020) increase the risk of globalization of RVFV.**

*** Balkhy HH, Memish ZA. Rift Valley fever: an uninvited zoonosis in the Arabian Peninsula. Int J Antimicrob Agents. 2003;21: 153–157.
* Eisa M, Kheir el-Sid ED, Shomein AM, Meegan JM. An outbreak of Rift Valley fever in the Sudan - 1976. Trans R Soc Trop Med Hyg. 1980;74: 417–419.
* LaBeaud AD, Muchiri EM, Ndzovu M, Mwanje MT, Muiruri S, Peters CJ, et al. Interepidemic Rift Valley fever virus seropositivity, northeastern Kenya. Emerg Infect Dis. 2008;14: 1240–1246.
* Meegan JM, Bailey CL. Rift Valley fever. In: Monath TP, editor. The Arboviruses. CRC Press, Inc., Boca Raton, FL; 1989. pp. 51–76.
* Mohamed AM, Ashshi AM, Asghar AH, Abd El-Rahim IHA, El-Shemi AG, Zafar T. Seroepidemiological survey on Rift Valley fever among small ruminants and their close human contacts in Makkah, Saudi Arabia, in 2011. Rev Sci Tech. 2014;33: 903–915.
* Napp S, Chevalier V, Busquets N, Calistri P, Casal J, Attia M, et al. Understanding the legal trade of cattle and camels and the derived risk of Rift Valley fever introduction into and transmission within Egypt. PLoS Negl Trop Dis. 2018;12: e0006143.
* Tigoi C, Sang R, Chepkorir E, Orindi B, Arum SO, Mulwa F, et al. High risk for human exposure to Rift Valley fever virus in communities living along livestock movement routes: A cross-sectional survey in Kenya. PLoS Negl Trop Dis. 2020;14: e0007979.**

We added three of the suggested citations lines 392-394: ‘Future efforts should focus on incorporating field constraints into their scenarios, while keeping in mind the transboundary nature of Rift Valley fever virus transmission [124–126].’

**Reviewer #2: The review is quite detailed. The gap in literature was pointed out. Below are some comments and suggestions,**

**1. Page 2, Line 4, the sentence `... go the extra miles' should be rephreased.'**

Done: ‘modellers need to improve their code accessibility’ line 40.

**2. Page 2, Line 43, remove `in'.**

Done.

**3. Page 3, Line 73, the sentence needs to be rephrased.**

We rephrased, lines 73-74: “Mathematical models are useful to project epidemiological scenarios, including control strategies. This can be done at large scales (temporal [24], spatial [25], or demographic [25])”.

**4. Page 3, Line 83, change `risk' into `risks'.**

Done: we changed to ‘model the risk of RVF occurrence in animals’

**5. Page 4, Line 93, remove `models in'.**

We changed into ‘knowledge gaps left unaddressed by models’.

**6. Page 6, Line 139, change `model' into `models'.**

Amended.

**7. Page 21, Line 333, change `was' into `were'.**

Done.

**8. Page 22, Line 342, change R0 into $R_0$.**

Done.

**9. Page 22, Line 354, add comma after `However'.**

Done.

**10.Page 23, Line 371, remove `between'.**

Done.

**11. Page 31, Page 32, add commas between the two equalities in equations (1), (2), and (3).**

We chose to write one equation per line to ease the reading.
