# Supplementary material for: Mechanistic models of Rift Valley fever virus transmission: A systematic review
Source: PLoS Negl Trop Dis. 2022 Nov 18;16(11):e0010339. doi: 10.1371/journal.pntd.0010339 (PMC9718419; doi:10.1371/journal.pntd.0010339)
Supplement: Attachment — Submitted filename: Response to reviewers v2.docx [file pntd.0010339.s003.docx]

**Dear Mr. Drouin,**

**Thank you very much for submitting a revised version of your manuscript "Mechanistic models of Rift Valley fever virus transmission dynamics: A systematic review" for consideration at PLOS Neglected Tropical Diseases. Based on the review, we are likely to accept this manuscript for publication, providing that you modify the manuscript according to my and the reviewer’s recommendations.**

**Below are a few more comments that need to be addressed.**

We would like to thank the editor for the attention given to our revised version and for the valuable comments. We respond to each of specific comments in the following section.

*Please note that lines numbers refers to the final version, without tracked changes.*

**Specific comments:**

**1. Line 59: Yes, RVFV has been detected in several Anopheles species, but are Anopheles competent vectors of RVFV. Just because a mosquito feeds on a viremic animal and ingests virus does not make it a vector. Anopheles tend to have an extreme salivary gland barrier for RVFV and are virtually unable to transmit this virus. While rarely tested for RVFV, various species of sand flies are competent vectors, and RVFV is a member of the genus of sand fly fever viruses**.

We agree with the editor on the fact that *Anopheles* species are not the most likely to have an important vector role, despite some primary evidence of the competence of field-caught *Anopheles coustani* in Madagascar (Nepomichene TNJJ, Raharimalala FN, Andriamandimby SF, Ravalohery JP, Failloux AB, Heraud JM, et al. Vector competence of *Culex antennatus* and *Anopheles coustani* mosquitoes for Rift Valley fever virus in Madagascar. Medical and Veterinary Entomology. 2018;32(2):259–62.). To avoid confusing the reader, and as *Culex* and *Aedes* are the two main vector genera, we simplified the sentence to:

“Transmission of Rift Valley fever virus (RVFV) mainly involves *Aedes* and *Culex* spp. mosquitoes [2], some of which are present in Europe and North America [3–10], but other genera may also be potential vectors [11–14].” (Lines 60-62)

We added two references of reviews of mosquitoe species potentially vector of RVFV:

13. Tantely LM, Boyer S, Fontenille D. A review of mosquitoes associated with Rift Valley fever virus in Madagascar. Am J Trop Med Hyg. 2015 Apr 1;92(4):722–9.

14. Lumley S, Horton DL, Hernandez-Triana LLM, Johnson N, Fooks AR, Hewson R. Rift Valley fever virus: strategies for maintenance, survival and vertical transmission in mosquitoes. J Gen Virol. 2017 May;98(5):875–87.

Regarding sand flies, please see our response to comment 6.

**2. Line 195, Table 1: What does “Vector health status” mean? Should that be “Vector infection status?”**

We changed both column names: “Host infection state”s and “Vector infection states”. We use “state” and not “status” to be consistent with the terminology classically used in epidemiological models, with compartment referred to as “states”.

**3. Line 283: Much better, but is “unrealistic” sufficient? Remember, The Romoser paper detected evidence of RVFV in one egg in 8.6% of the RVFV-inoculated mosquitoes. Because they were inoculated, this rate would be much higher than in mosquitoes that had ingested RVFV. Also, given that each mosquito lays about 150 eggs/oviposition cycle, the 8.6% of females with an infected egg translates to 0.06% of the progeny of the RVFV-inoculated mosquitoes would be infected. If the model used 20%, then to me, the model is beyond unrealistic when the rate would be <0.1% of the progeny of RVFV-inoculated mosquitoes. However, as you now point out, if a mosquito had a stabilized infection with RVFV, that would change everything.**

We have changed our sentence, lines 282-283: “Such a rate seems much higher than what has been observed experimentally [112, 113].”

We also added as reference 113:

Linthicum KJ, Davies FG, Kairo A, Bailey CL. Rift Valley fever virus (family Bunyaviridae, genus Phlebovirus). Isolations from Diptera collected during an inter-epizootic period in Kenya. Journal of Hygiene. 1985;95(1):197–209.

**4. Lines 304-306: Yes, the importation of an infectious disease into an area where it is not currently present can allow for a major outbreak. Why is that surprising? If the Ro is > 1, then you will get an outbreak. For RVF, I believe that the Ro is strongly affected by the presence (and number) of competent vectors and the presence of competent vertebrate amplifying hosts (e.g., cattle, goats, sheep, and various wildlife).**

Although it might not seem surprising conceptually, we would argue that the added value of such a model (Gao et al. 2013) can be to quantify the threshold dynamics of the system. Moreover, the study by Gao et al. 2013 is more sophisticated than that, as it shows that due to animal movements, the disease can persist in patches with R_0_ < 1 if animals are introduced from a patch with R_0_ > 1, depending on the travel speed of animals across the patch.

We did not add this information to the manuscript in order to keep model descriptions / comparisons short and readable. A review cannot provide readers with all the details of all included papers, and we had to choose the key information.

**5. Lines 308-312: The association between bovine tuberculosis and RVF is interesting. Just curious, does infection with RVF make a cow more susceptible to BTB, or are herds that are in certain locations more like to be infected with both agents (i.e., neither pathogen affects the replication of the other, but certain areas are more likely to have either both or neither of the two pathogens)? Yes, if your model incorporates increases the amount of RVF with increasing amounts of BTB, isn’t it obvious that it will predict more RVFV in a population with a higher BTB positivity?**

In this study, the authors aimed to “determine whether animals with BTB were more or less likely to become infected with RVF”, considering that *M. bovis* infection leads to modification of the host immune system. They compared the effect of the 2008 outbreak in Kruger National Park among captive and free-ranging buffaloes (BTB+ and BTB- buffaloes were kept in the same location). The risk of being infected by RVFV was higher in BTB+ populations, as was the risk of abortion due to RVF.

We agree with the editor that an increase in RVF cases was certainly expected in this study, but the model can be helpful to quantify the impact of the association between BTB and RVF. Moreover, the authors showed that the number of RVF cases also increases in BTB- buffaloes, which was not necessarily expected. They also highlighted that the relationship between the increase in BTB prevalence and the number of RVF cases is nonlinear, as is the relationship between BTB prevalence and the time to peak of RVF cases and between BTB prevalence and the outbreak duration. We modified the sentence to incorporate these last two points:

“Once this effect was incorporated in a model, an increase in BTB prevalence nonlinearly affected three RVF outbreak metrics: the outbreak size in both BTB-infected and BTB-free populations, the timing of the peak, and the outbreak duration.”(Lines 311-314)

**6. Lines 312-317: Again, better, but why even include a paper about ticks? Because RVFV is a member of the genus Phlebovirus (i.e., sand fly fever virus) and various species of sand flies have been proven (in the laboratory) to be competent vectors of RVFV, why hasn’t someone included them in the model, or in the case of this manuscript, why isn’t it pointed out that these models are missing? To me, that is much more important than devoting space to a tick model.**

We included the study of Pedro et al. (2017) because it has been selected according to our inclusion criteria (mechanistic models with numerical application to RVFV). This paper considered not only *Hyalomma* ticks but also mosquitoes.

Regarding the phlebotomine sand flies, we added a sentence to account for the possible role of other biological and mechanical vectors and the fact that they are not taken into account in modelling studies:

“By contrast, other species which have been experimentally demonstrated as competent, either as biological (such as sand flies [116,117]), or mechanical vector [118] have not been included so far in RVFV models.” (Lines 319-321)

This adds 3 references:

116.	Dohm DJ, Rowton ED, Lawyer PG, O’Guinn M, Turell MJ. Laboratory transmission of Rift Valley fever virus by *Phlebotomus duboscqi*, *Phlebotomus papatasi*, *Phlebotomus sergenti*, and *Sergentomyia schwetzi* (Diptera: Psychodidae). J Med Entomol. 2000 May 1;37(3):435–8.

117.	Turell MJ, Perkins PV. Transmission of Rift Valley fever virus by the sand fly, *Phlebotomus duboscqi* (Diptera: Psychodidae). Am J Trop Med Hyg. 1990 Feb 1;42(2):185–8.

118.	Hoch AL, Gargan TP, Bailey CL. Mechanical transmission of Rift Valley fever virus by hematophagous diptera. Am J Trop Med Hyg. 1985 Jan 1;34(1):188–93.

**7. Lines 363-363: How the use of larvicides, even in a low transmission context could increase RVF incidences seems contra intuitive to me. How did this model find this increase in incidence? Yes, during an outbreak, larvicides would be ineffective as they would not reduce the number of infected vectors present. However, the use of adulticides would reduce the vector population and be more effective. So, if you compared areas that used larvicides with those that used adulticides, then there might be more transmission in the areas that used larvicides. However, that is not what the manuscript implies.**

Gaff et al. (2011) suggest that the deleterious effect of larvicides (in low transmission context only), might be due to the reduction in adult mosquito population renewal, which in turn increases the proportion of infected among adult mosquitoes. In this model, the force of infection chosen is infectious frequency dependent. This means that the number of new infections in hosts is only driven by the proportion of infectious mosquitoes in the population, and that the contact rate is otherwise constant, thus not affected by the vector-to-host ratio (which is decreased by the use of larvicides). This result is therefore likely to be strongly influenced by this modelling choice, as some other results presented in the review. We think that letting the section on the force of infection at the end of the paper is still relevant, not to drown non-modeller readers in technical terms, but we emphasize again at the end of our introduction, lines 108-109: “In that regard, we therefore insist that key results presented in this review should be interpreted with this methodological choice in mind.”

We also added an explanation at the end of the sentence, lines 367-371:

“In one study [62], all strategies were efficient to reduce the number of cases when compared in a context of high virus transmission, but using larvicides in a low transmission context led to an increased incidence, due to the subsequent increase in the proportion of infected mosquitoes and the choice of an infectious frequency dependent force of infection (Box 1).”

**8. Lines 379-383: Yes, I know that the model predicts that “stamping out in a 20km radius was the most effective strategy. However, given that this disease has never been observed in Europe, how accurate is that model? Would intensive mosquito control be critical. How many infectious cases in cattle would be subclinical and allow the virus to continue to spread? My concern is that given the lack of data going into these models, that a control practice based solely on this model may allow for RVFV to become enzootic.**

Obviously, simulating an outbreak in a currently RVF-free zone relies on a lot of “what if”, and we have virtually no idea of how accurate this model could be. But it is still a worthy exercise to prepare such scenarios to the best of our ability given current knowledge. At no point do we say that this model should form the basis of a control strategy. We have repeatedly highlighted the lack of field data which prevents models from approaching operational scenarios, even in zones with recurring RVFV circulation.

The authors did not test vector control scenarios in their model applied to the Netherlands, so their results are assumed to be valid for “stamping out” only situations. Regarding subclinical cases, the authors did not take them directly into account, but did focus on early detection and surveillance, and assumed that high mortality rates in juveniles and abortion storms could quickly signal the presence of RVFV. We did not add this information to the manuscript in order to keep model descriptions / comparisons short and readable. However, we changed our sentence, lines 386-389: “In the Netherlands, a RVFV-free area, a model concluded that stamping out in a 20 km radius around an outbreak could be the most effective strategy, when comparing with scenarios of vaccination or other culling strategies [78 – Model 2].”

**9. Line 478: Yes, the models only applied to “livestock,” but is that really on a single vertebrate host species? Livestock consists of several species.**

We clarified, line 484 “a single vertebrate host category”.

**10. Line 512-516: Thanks for modifying this a little, but it illustrates a much bigger problem. Yes, many studies consider humans to be a dead-end host. This is probably because with nearly all of the other zoonotic arboviruses, i.e., eastern equine encephalitis virus, western equine encephalitis virus, Japanese encephalitis virus, etc., humans produce a very low viremia and are thus a dead-end host. Therefore, humans must be a dead end host for RVF too. However, studies indicate that the viremia in humans infected with RVFV can be extremely high, often >8 logs, i.e., higher than in adult cattle, goats, or sheep (see Meegan 1979, Trans R Soc Trop Med). Note that the de St. Maurice et al. 2017 paper cited (ref 139), only found viremias about 5-6 logs, but didn’t start testing until about 5 days after the onset of fever, by which time viremias would have fallen greatly. Yes, unfortunately, there are relatively few studies that have looked at viremias in humans, but even implying that humans are a dead-end host is misleading and dangerous. Given the number of humans that visit enzootic areas every year, if one of them became infected, given rapid transportation, they could easily transport RVFV back to an area (Europe or the Americas) where this virus is not yet present. Transportation of animals is important for local movement of RVF, but humans may be much more important for long range movement.**

We thank the editor for the interesting sharing on that subject. We fully agree on the fact that the viremia in some human individuals could be sufficient to infect mosquitoes, as demonstrated in the studies on that matter. We also fully agree that there are few studies concerning the human viremia and the ability to transmit the virus to mosquito, and that this is an important knowledge gap.

We believe that our paragraph reflects the actual state-of-the-art by stating that: 1) in literature, authors mainly consider humans as dead-end hosts; 2) contradictory data suggest the possibility for humans to develop a sufficiently high viremia to infect mosquitoes; and finally 3) modelling studies could be helpful to build on this hypothesis and to test scenarios.

We added the suggested reference in the sentence as reference 146, line 519 (Meegan JM. The Rift Valley fever epizootic in Egypt 1977–1978 1. Description of the epizootic and virological studies. Trans R Soc Trop Med Hyg. 1979 Jan 1;73(6):618–23.). We also modified the last sentence of the paragraph:

“Considering this knowledge gap and the difficulty to obtain direct observations on that matter, it would seem relevant for future models to evaluate whether human-to-mosquito transmission is necessary to explain observed transmission dynamics.” (Lines 520-522)

**11. Lines 551-553: See also Rossignol PA, Ribeiro JM, Jungery M, Turell MJ, Spielman A, Bailey CL. Enhanced mosquito blood-finding success on parasitemic hosts: evidence for vector-parasite mutualism. Proc Natl Acad Sci U S A. 1985 Nov;82(22):7725-7. That paper showed that mosquitoes were able to feed significantly more rapidly on a RVFV-infected animal as compared to an uninfected one and is one of the explanations for why mosquitoes are more successful in feeding on a RVFV-infected animal. Not mentioned here is that infected animals may have less “anti-mosquito” behavior because they are ill or that because they are febrile that they might be more “attractive” to a host-seeking mosquito.**

This article has already been included in the references of the aforementioned sentence (previously as reference 146, and as reference 153 in the revised version of the manuscript):

“However, studies have suggested that the infected or uninfected status of the host might also play a role, for different pathogens [149,150], including for RVFV [152,153].” (Lines 559-561).

**Minor:**

**12. line 40: “modellers…” should be “modelers…”**

We changed and used the spelling “modeling” and “modelers” in the whole manuscript.

**13. Line 43: There should not be a “,” after “priorities” as the next phrase is not a complete sentence. Same comment on line 51 for the comma after “choices.”**

This has been modified.

**14. Line 289 (and elsewhere): Is it “endemic” or “enzootic” circulation/areas?**

As RVF is a zoonotic disease, we chose the word “endemic” as a default in general sentences, without trying to determine for each context the enzootic vs endemic nature of the circulation. However, for cases describing models including only animal hosts, we now use enzootic. Therefore, the occurrence of “endemic” line 288 has been modified to “enzootic”, as suggested. The other occurrences of the two words have been checked according to this criterion.

**15. Line 346: What do you mean by “both host populations?” Is it cattle and small ruminants or is it cattle and humans? Above the sentence seems to imply small ruminants, but the discussion below implies humans.**

We changed the words “host populations” to “ruminant populations” to be more explicit:

“Regarding the choice of hosts to vaccinate, Gachohi et al. (2016) [96] highlighted that while small ruminants needed a smaller coverage than cattle to achieve a given reduction in incidence, the vaccination of cattle provided the benefit of protecting both ruminant populations.” (Lines 348-350)

To avoid any further confusion, we also clarified line 352-354: “Métras et al. (2020) [76] was the only model evaluating a possible human vaccination campaign. They estimated that, in the context of Mayotte island, vaccination of livestock was the most efficient strategy to limit human cases, compared to human vaccination.”

**16. Line 394: Shouldn’t “Rift Valley fever virus transmission” be “RVFV transmission?**

“Rift Valley fever virus” has been replaced by “RVFV”.

**17. Line 452: Shouldn’t “exposure ; and” be “exposure; and” with no space before the colon?**

The space before the semi-colon has been removed.

**18. Line 610: As “years” are a unit of measurement, it should be “5 years…” Similarly, on line 611, it should be “10 previous years…”**

This has been modified.

**19. Line 640: Shouldn’t “Bibliography” be “References?”**

This has been modified.

**20. References: Much better.**

**a. Line 735, reference 40: “Rift Valley Fever…” should be “Rift Valley fever…?” See also references 76, 93, 125 and possibly others.**

**b. Line 740, reference 42: Here, “Culex pipiens” is not in italics, but in the other references, species names have been in italics. Please be consistent.**

**c. Line 1014, reference 145: Only the first word and proper nouns in a title should be capitalized.**

All the suggested modifications to references have been changed.

**Reviewer #3: This study represents a valuable exercise in reviewing all modeling studies of RVFV using a systematic literature review. Although reviews of the RVFV system have occurred frequently, this study updates a synthesis of published models and provides an excellent framework for evaluating different aspects of past applied and theoretical models. The inclusion of inheritance connections among the studies is also a nice feature. I have a few minor observations to improve the manuscript.**

We are thankful to the reviewer 3 for the careful reading and the positive comments.

**Ln. 144. Could ‘and insights remain distant from field preoccupations’ be explained better as I don’t understand what that means**.

We clarified, lines 144-148 “Grey models are those which do not fit into these well-defined categories. In some cases, authors do not use data but demonstrate a strong will to adapt their models to a specific geographical or epidemiological context. In other cases, despite the use of data, the model developed is still very conceptual and lacks realism in its key features. In such cases, the model analysis rarely deepens the epidemiological understanding of the pathosystem.”

**Ln. 417-475. This is a great observation to point out. However, every author on this current systematic review appears to have an affiliation with an institution in France.**

We think our comment on including authors from local research institutes is mostly relevant to studies presenting original research results (with new data, or in a specific geographical context). We clarified, lines 480-482: “This is important to develop more realistic and useful models, and at a time when concerns are being raised about the equity of South-North research collaborations [135,136].”

Our work is a systematic review. Yet, we would like to point out that models published by the authors from this systematic review did follow this rule:

Métras et al. 2017 and 2020 in Mayotte

Durand et al. 2020 (B. Durand and V. Chevalier in the authors) in Senegal

Cecilia et al. 2020 in Senegal

Cavalerie et al. 2015 (P. Ezanno in the authors) in Mayotte

Tennant et al. 2021 (R. Métras in the authors) in the Comoros

Nicolas et al. 2014 (B. Durand and V. Chevalier in the authors) in Madagascar

This long history of collaboration with scientists from African or Indian Ocean countries is in our opinion sufficient to ensure that this systematic review reports and criticizes existing work with field preoccupations in mind.

**Ln. 518. This statement ‘while models with one taxon often did not precise the genus of vector studied’ could be changed to ‘while models with one vector taxon did not specify genus or species’ for clarity.**

This has been modified to: “while models with one vector taxon often did not specify the genus or species (10/20).” (Lines XXX)

**Figure 2 and 5. The legend nor text explain what ‘FR’, ‘FI’, mean in either of these figures. The Box explains but it would be helpful to at least include the name of the abbreviation to help interpret the figures**.

Significations of abbreviations have been added to the figures’ captions.

**Figure 4. For the ‘Location and number of RVF models.’. Does this ‘location’ refer to where the authors were from or where the model was parameterized in terms of geographic location? I assume it is the latter but it is hard to tell this in the methods or this Figure 4 legend so this could be clarified.**

In this figure, “location” refers to the geographical context of the model, and not to the geographical origin of the authors. We changed the word “location” to “geographical context” in the first sentence of the figure caption to be more explicit:

“Figure 4 – A - Geographical context and number of RVF models. Grey models are mapped but not counted in totals because they sometimes refer to a non-precise context (e.g., East Africa, North America, see B). Locations of grey models which are not also studied in applied models are shown in grey (Egypt, Sudan). The point north of Madagascar, accompanied by text, is centered on the Comoros archipelago. It stands for 4 models applied to Mayotte island and 1 model applied to the whole Comoros archipelago, including Mayotte. B - Scale of applied and grey models. Labels represent model locations, with one label per model, hence sometimes repeated locations. Labels are colored to help identify the scale (y-axis). East African Community = Burundi, Kenya, Rwanda, and Tanzania. Besides, all 4 models applied to Mayotte considered the whole island (374 sq. km), but those models are classified as sub-national. Sub-figures A and B are not restricted to spatialized models.” (Lines 434-441).

Additionally, we would like to point out some modifications that we have made independently of the editor and reviewers’ comments:

- We removed the term “dynamics” from the title.

- We slightly changed the abstract to make it more precise and appealing

- We clarified a modeling result lines 305-308.

- We split a paragraph in two to better highlight the lack of data on vector population dynamics, lines 552-557

- We changed the figures inserted in the manuscript file to reflect the .tiff files uploaded to the submission system.

- We added a Github repository link in the Supporting information to access to the code used to produce figures and summary statistics:

“Code use to produce figures and summary statistics is available in Github public repository at <https://github.com/helenececilia/riftvalleyfever-model-review.git> . (Lines 648-649)”
