# Supplementary material for: Mechanistic models of Rift Valley fever virus transmission: A systematic review
Source: PLoS Negl Trop Dis. 2022 Nov 18;16(11):e0010339. doi: 10.1371/journal.pntd.0010339 (PMC9718419; doi:10.1371/journal.pntd.0010339)
Supplement: Attachment — Submitted filename: Response to reviewers v4.docx [file pntd.0010339.s004.docx]

**Thanks for the revised version and addressing the comments made by me and the reviewer. My only real issue is stating that people consider humans to be a dead-end host for RVFV. Please see my comment 5 below as well as a few minor comments.**

**1. Line 33: Insert a space between “models” and “with”**

Done.

**2. Line 39: Insert a space after the period after “transmission” and shouldn’t this be in the past tense, so shouldn’t “we note a…” be “we noted a…?”**

We added a period and changed to past tense.

**3. Line 193: Shouldn’t there be a “period” after “et al” instead of a “comma?”**

We changed the comma to a period. We also checked all occurrences of “et al.,” in the manuscript and changed it to “et al.” when in the middle of a sentence.

**4. Lines 370-372: Again, how could the use of larvicides increase the proportion of infected mosquitoes? Yes, the use of larvicides is generally ineffective in disease prevention. This is because it does not kill any of the infectious mosquitoes that are already out there. Was this only in their model, or is there any real-world evidence that the use of larvicides led to an increased evidence of disease.**

In this study, the use of larvicides reduces the renewal of the adult mosquito population. All other parameters remain constant compared to the reference scenario (same vector-host contact rate, same adult mosquito mortality etc.). Therefore, this adult mosquito population, while being poorly renewed, continues to get infected by biting infectious livestock, resulting in a higher proportion of infected adults mosquitoes, at least transiently. Then, due to the functional form chosen for the force of infection (proportional to the number of infected mosquitoes but not to the vector-to-host ratio), the incidence increases in hosts.

But this result is indeed counter-intuitive biologically and risks confusing the reader. As it is driven by the choice of the functional form for the force of infection, which is not justified in the paper, and that no field data are used to back up this pattern, we think it is judicious to remove this sentence not to put an unwarranted emphasis on it.

**5. Lines 519-523: Yes, there are a number of papers that have mentioned that humans are a dead-end host for RVFV. Note, if this paper is publish as currently written, it too will be cited as evidence that humans are a dead-end host for RVFV. They are not! Unfortunately, as humans are dead-end hosts for most zoonotic viruses (West Nile, eastern equine encephalitis, Japanese encephalitis, St. Louis encephalitis, etc.) some people assumed that humans must be a dead-end host of this zoonotic pathogen. In the very few studies that have looked at viremias in active human infections, viremias can be very high (actually higher than in cattle or sheep). That being said, humans probably play a very small role in the outbreak spread of RVFV because the reduced number of mosquito bites/day on a human compared to a cow. However, that small role is not due to them being a dead-end host, which implies that the viremia is too low to infect a mosquito. In fact, humans are probably the most likely way that RVFV will eventually spread to the Americas and Europe, i.e., in a tourist bitten by an infectious mosquito while visiting one of the enzootic areas to look at wildlife. I do not want this paper to be another one cited that humans are a dead-end host. How about, “…human-to-mosquito transmission. This is in line with humans not being considered important in the outbreak spread of RVFV because of limited mosquito biting of humans compared with livestock. However, some…”**

We agree and rephrased the paragraph to improve clarity in lines 516-523:

“From these ten models, three [71,93,102] considered human-to-mosquito transmission. This low representation of this transmission route may reflect a confusion in the likely small role played by humans in RVFV transmission. As human-mosquito transmission has not been documented so far, humans may often be mistakenly considered as dead-end hosts [20,143]. Nevertheless, some data, while scarce, suggest humans could develop a high viremia [143–146] which would be sufficient to infect mosquitoes. Under this hypothesis, humans could have a role in the long distance spread of the virus [148]. Considering this knowledge gap and the difficulty to obtain direct observations on that matter, it would seem relevant for future models to evaluate whether human-to-mosquito transmission is necessary to explain observed transmission dynamics.”

**6. Line 550: Should “genus” be “genera” as it could be more than one genus?**

This was indeed a mistake, and it has been changed.

Additionally, we would like to highlight that figure 2 has been changed to add a parental connection between two papers, and we adapted the text to take this change into account.
